# High Stability of Methanol to Aromatic Conversion over Bimetallic Ca,Ga-Modified ZSM-5

**DOI:** 10.1021/acscatal.1c05481

**Published:** 2022-02-23

**Authors:** Chuncheng Liu, Evgeny A. Uslamin, Elena Khramenkova, Enrico Sireci, Lucas T. L. J. Ouwehand, Swapna Ganapathy, Freek Kapteijn, Evgeny A. Pidko

**Affiliations:** † Inorganic Systems Engineering, Department of Chemical Engineering, Delft University of Technology, Van der Maasweg 9, 2629 HZ Delft, The Netherlands; ‡ Radiation Science and Technology Department, Delft University of Technology, Mekelweg 15, 2629 JB Delft, The Netherlands; § Catalysis Engineering, Department of Chemical Engineering, Delft University of Technology, Van der Maasweg 9, 2629 HZ Delft, The Netherlands

**Keywords:** methanol-to-aromatics, bimetallic catalyst, dehydrogenation, global optimization, catalyst deactivation

## Abstract

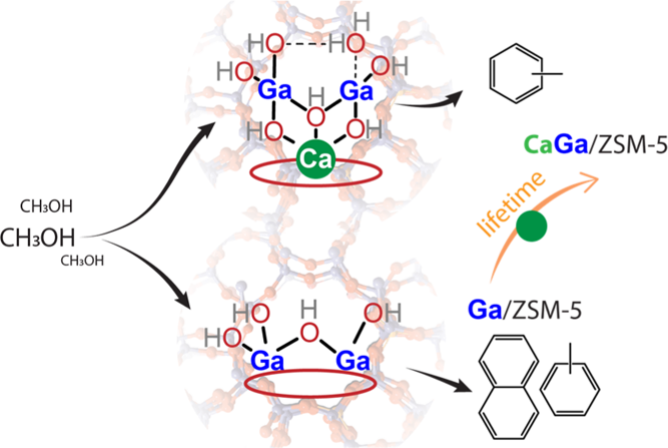

The production of valuable aromatics and the rapid catalyst deactivation due to coking are intimately related in the zeolite-catalyzed aromatization reactions. Here, we demonstrate that these two processes can be decoupled by promoting the Ga/HZSM-5 aromatization catalyst with Ca. The resulting bimetallic catalysts combine high selectivity to light aromatics with extended catalyst lifetime in the methanol-to-aromatics process. Evaluation of the catalytic performance combined with detailed catalyst characterization suggests that the added Ca interacts with the Ga-LAS, with a strong effect on the aromatization processes. A genetic algorithm approach complemented by ab initio thermodynamic analysis is used to elucidate the possible structures of bimetallic extraframework species formed under reaction conditions. The promotion effect of minute amounts of Ca is attributed to the stabilization of the intra-zeolite extraframework gallium oxide clusters with moderated dehydrogenation activity.

## 1 Introduction

Aromatic compounds, namely, benzene, toluene, ethylbenzene, and *o*-/*m*-/*p*-xylenes (BTEX), are the basic platform chemicals for the production of polymers, coatings, solvents, and other functional materials.^1−3^ Currently, the production of aromatics solely relies on fossil feedstocks such as the naphtha steam cracking. The methanol-to-aromatics (MTA) process is a promising route to meet an ever-increasing demand in sustainable BTEX. Methanol (MeOH) is readily available from various sources including shale gas, biomass, and CO_2_.^4−8^

MeOH conversion by zeolite catalysts proceeds via a complex network of transformations, involving the cooperation between zeolite Brønsted acid sites (BASs) and confined hydrocarbon intermediates, commonly referred to as the hydrocarbon pool.^9−13^ A dual-cycle mechanism was proposed to describe the conversion of MeOH over H-ZSM-5 where light olefins are formed from the methylation/cracking of the longer olefins, while BTEX and ethylene originate from the alkylation/dealkylation process of methylated aromatic species.^14^

A typical strategy to increase the selectivity to aromatics in MeOH conversion involves zeolite modification by Zn,^15−22^ Ga,^23−30^ and Ag.^31,32^ The aromatization process is then a result of the direct dehydrogenation reactions catalyzed by the formed Lewis acid sites (LASs). However, the increase in selectivity to aromatic intermediates accelerates the formation of the polycyclic aromatics causing catalyst deactivation.^26,29,33−38^ To improve the catalyst stability, several approaches have been proposed. For instance, hierarchical mesoporous materials^39^ and nanosized zeolites^40^ can be used to tune the diffusion of the reaction products from zeolite micropores. Postsynthetic modifications such as mild steaming^41^ are employed to adjust the acidic properties of zeolite. Modifications of the entire process such as H_2_ co-feeding could suppress the transformation of the active intra-zeolite species to polycyclic aromatics coke precursors.^42^ Recent studies reveal that the catalyst deactivation can be moderated by the presence of Ca-LAS in the methanol-to-olefin process.^43^ However, most of the methods targeting the extended process lifetime result in a significant decrease in aromatics selectivity.

Herein, we present the investigation of the catalytic properties of bimetallic [Ca,Ga]/H-ZSM-5 with improved stability and high yield of BTEX in the MTA process. The structural and acidic properties of Ca-doped Ga/H-ZSM-5 were evaluated by X-ray diffraction, N_2_ physisorption, and FTIR spectroscopic analysis of pyridine adsorption. To figure out the mechanistic basis for the catalytic effect of Ca addition, the computational modeling was employed. A genetic algorithm approach was used to determine the structures of intra-zeolite metal clusters, followed by the ab initio thermodynamic analysis to assess their stability under the catalytically relevant conditions. The reactivity of the most stable configurations was probed using ethane dehydrogenation as a model reaction.

## 2 Methods

### 2.1 Catalyst Preparation

Protonic H-ZSM-5 (CBV5020E) with a Si/Al ratio of 25 was purchased from Zeolyst Int. and denoted as H-ZSM-5. Ga-modified ZSM-5 sample was prepared via incipient wetness impregnation with an aqueous solution of Ga(NO_3_)_3_ [gallium(III) nitrate hydrate, Sigma-Aldrich, 99.9% trace metals basis]. After the impregnation, the sample was first dried at 80 °C overnight and then calcined at 550 °C (ramp rate 2 °C/min) under static air for 6 h. To increase the dispersion of Ga species in the zeolite micropores, the calcined Ga/H-ZSM-5 sample was further reduced at 500 °C (2 °C/min) in a 30 vol % H_2_ in Ar flow for 7 h. After that, the sample was cooled to 150 °C and re-oxidized in a flow of air for 1 h.^37^ Samples containing 1, 2, and 3 wt % Ga were prepared, denoted as Ga(*x*) (*x* = 1, 2, or 3) where the value in the bracket represents the weight loading of the metal. Ca(0.02) and Ca(1) samples, containing 0.02 and 1 wt % Ca, respectively, were prepared via the same incipient wetness impregnation procedure with calcium nitrate tetrahydrate solutions, followed by calcination at 550 °C (ramp rate 2 °C/min) under static air for 6 h without the final reduction–oxidation step.

A second incipient wetness impregnation was carried out with the prepared Ga(2) to obtain the bimetallic catalyst. Aqueous solutions with different concentrations of Ca(NO_3_)_2_ (calcium nitrate tetrahydrate, Sigma-Aldrich, ACS reagent, 99%) were used. After impregnation, the as-prepared sample went through drying and calcination steps under the same conditions as described above. The notation is Ca(*x*)Ga(2) where *x* represents the wt % loading of Ca. To check the relevance of the addition order, one sample was prepared according to the above procedures in which the Ca was added first and then the Ga. This sample is denoted as Ga(2)Ca(0.02).

### 2.2 Catalytic Tests

MTA catalytic runs were performed at 450 °C using a fixed-bed reactor setup. In a typical experiment, a 4 mm (ID) quartz reactor tube was filled with 40 mg of sieved zeolite fraction (particle size 150–212 μm). MeOH was fed into the reactor through a thermostatted saturator with liquid MeOH (Sigma-Aldrich, for HPLC, ≥99.9%) using N_2_ as a carrier gas. The reaction products were analyzed with an online Thermo Trace GC (Trace 1300 Ultra, Thermo Fisher) equipped with a thermal conductivity detector (TCD) coupled with a PoraPLOT Q precolumn (2 m; i.d. 0.32 mm; film thickness 20 μm) and Molsieve 5 Å column (10 m; i.d. 0.32 mm) for the analysis of permanent gases, a flame ionization detector (FID) equipped with RTX-1 column (2 m; i.d. 0.32 mm; film thickness 5 μm), and an Al_2_O_3_/KCl column (15 m; i.d. 0.32 mm; film thickness 10 μm) for the analysis of C_1_ to C_4_ hydrocarbons and another FID equipped with a RTX-VMS column (30 m; i.d. 0.33 mm; film thickness 3 μm) for C_5+_ hydrocarbons.

Prior to the reaction, the catalyst was activated in 50 mL/min air up to 550 °C (5 °C/min) for 1 h and then cooled down to the reaction temperature of 450 °C. The initial partial pressure of MeOH in the feed flow was set at 5.2 kPa. The corresponding WHSV amounted to 5.3 g_MeOH_ g_cat_^–1^ h^–1^. The MeOH conversion (excluding DME), reaction selectivity, and yield were calculated on a carbon molar basis as follows
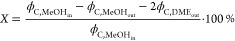
1
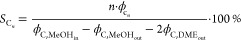
2
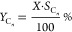
3where *X*, *S*_C_*n*__, and *Y*_C_*n*__ represent the conversion of MeOH and dimethyl ether, carbon selectivity of certain hydrocarbon products, and the corresponding carbon yield in the exhaust with a carbon number equal to *n*, respectively.

### 2.3 Catalyst Characterization

The elemental composition of each sample was assessed with inductively coupled plasma atomic emission spectrometry (ICP-AES) using a Perkin Elmer Optima 5300DV instrument (glass torch + Sapphire injector). Before measurement, ca. 50 mg of the sample was digested in an aqueous solution of 4.5 mL 30% HCl + 1.5 mL 65% HNO_3_ + 0.2 mL 40% HF using a microwave heater operating at maximal power for ca. 60 min. The resulting solutions were then diluted to 50 mL with deionized water.

The X-ray powder diffraction (XRD) patterns were obtained in Bragg–Brentano geometry with a Bruker D8 Advance X-ray diffractometer using monochromatic Co Kα (λ = 1.788970 Å) radiation between 2θ = 5 and 55°. XRD was measured for all catalysts after the final calcination. The patterns were analyzed by parametric Rietveld refinement^44^ using TOPAS (Topas Academic V6, Bruker AXS GmbH) to extract the unit cell parameters (orthorhombic cell; *Pnma* space group) in the MFI framework. The crystal size analysis was carried out by applying the Scherrer method
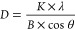
where *D* represents the diameter of a spherical nanocrystal with *K* = 0.89, λ is the wavelength of X-ray, θ is the diffraction angle of the band at 9.1° (*hkl* = 101), and *B* is the corrected half width of the observed half width considering the instrumental impact.

N_2_ physisorption analysis was performed to evaluate the microporous properties of each sample using Tristar II 3020 at −196 °C. Prior to measurements, samples were dried and degassed at 350 °C for 6 h under constant N_2_ flow.

Transmission FTIR spectroscopy of adsorbed pyridine (anhydrous, Sigma-Aldrich, 99.8%) as a probe molecule was used to accomplish the acidity characterization. Sample (20 mg) was pressed into a self-supporting wafer with a diameter of 1.6 cm and then placed in an IR quartz cell. Before pyridine adsorption, the specimen was activated at 400 °C (1 °C/min) for 7 h under vacuum and then cooled down to room temperature. Pyridine vapor was dosed in the IR cell via a separate chamber containing pyridine with a known volume and pressure. The specimen was then heated at 160 °C to allow the sufficient diffusion of the probe molecule for 1 h and then cooled down to room temperature for spectra collection. The spectra were recorded using a Nicolet 6700 FT-IR (Thermo Scientific) at 2 cm^–1^ resolution equipped with an extended KBr beam splitting and an MCT detector. The amount of BAS and LAS was derived from the absorbances at 1545 and 1458–1446 cm^–1^ using the integrated molar extinction coefficients of 0.73 and 1.11, respectively.^45^ Assuming that one pyridine molecule is only adsorbed on one BAS/LAS, the following equations were used to estimate *C*_BAS_ and *C*_LAS_
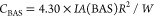
4
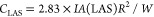
5where *IA* (BAS, LAS) represents the integrated absorbance of the band at 1545 and 1458–1446 cm^–1^, *R* is the radius (cm), and *W* is the weight of the self-supporting sample wafer (g).

For FTIR spectroscopy with adsorbed acetonitrile-*d*_3_ (CD_3_CN, Sigma-Aldrich, ≥99.8 atom % D), the same wafer was prepared and then pretreated under the same conditions as described above. CD_3_CN vapor was dosed in the IR cell. IR spectra were recorded continuously at RT until saturation (CD_3_CN ∼2 mbar).

For 3-methylpentane (3-MP) cracking tests, 20 mg of catalyst (150–212 μm) was pretreated at 550 °C in 50 mL/min air prior to the reaction at 400 °C. 2,4-Dimethyl quinoline base was added in flow to deactivate surface acid sites.^46^ The partial pressure of 3-MP in 50 mL/min N_2_ was adjusted to control the total conversion of 3-MP below 10%. Besides the formed H_2_ as a side product of direct dehydrogenation over Ga-LAS, hydrogen, methane, and ethane are also selectively formed through the monomolecular cracking of the pentacoordinated carbonium ion formed by the protonation of the 3-MP molecule on the BAS. Moreover, the energetically favorable bimolecular cracking (H-transfer reaction)^47^ via primary carbenium ions forms mainly only hydrocarbons beyond C3.

### 2.4 Computational Modeling

The stability and reactivity of extraframework cations in cation-modified ZSM-5 zeolites were computationally studied using the cluster modeling approach. 22T cluster models representing the different environments of the alpha, beta, and gamma sites of ZSM-5 were constructed to accommodate the cationic ensembles. For each ZSM-5 cluster model, two Si^4+^ atoms were substituted with two Al^3+^ generating a negative charge in the system, which was compensated by extraframework oxygenated Ga or Ca–Ga cationic clusters. A Ga/Al ratio of 1 was assumed for all models. The −OH dangling bonds were used to terminate the cluster models. Varied chemical compositions of the cluster models were considered and the preferred structures were determined by using a fully automated genetic algorithm optimization strategy. The relative stabilities of the extraframework species with different stoichiometries under the catalytically relevant conditions were evaluated using the ab initio thermodynamic analysis.

Generic algorithm (GA) applies the principles from evolutionary biology by learning the structural features of a “good” solution throughout the operations of fitness assignment, crossover, mutation, and selection.^48,49^ In this study, the GA was executed and controlled using the Atomic Simulation Environment (ASE) employing a semi-empirical tight-binding calculator GFN1-*x*TB.^50−54^ A GA developed by Vilhelmsen and Hammer was utilized.^54^ The whole zeolitic framework was kept fixed during the GA runs. The workflow of GA starts by initializing a population consisting of 20 structures in random arrangements. The operation of selection uses an energy-based fitness function to rank the candidates, and the crossover operator picks the candidates as parents for new structure generation. The mutation probability was set to a 30% rate with equal probabilities for mirror and rattle mutations. The candidates were found to be converged as the maximum energy difference, the maximum interatomic distances and the maximum difference in interatomic difference reached 0.02 eV, 0.015, and 0.7 Å, respectively. In each run, the maximum number of cycles given to the algorithm to converge was 120. The calculation was considered to have converged if no significant change was recorded in the last five generations. The global minima for each stoichiometry are provided in the Supporting Information.

The global minima obtained from the GA runs were further optimized using the PBE-D3(BJ)^55−59^ (level of theory implementing a modified version of the mixed Gaussian and plane-wave code CP2K/Quickstep^60−64^). Using this method, the electronic charge density is calculated using plane waves, while the Kohn–Sham orbitals get extended in contracted Gaussians. A Gaussian basis set DZVP-MOLOPT-GTH basis was used,^65^ and the density cutoff of 280 Ry was employed. The Goedecker–Teter–Hutter pseudopotentials^66^ with a combination of nonperiodic wavelet-based Poisson solver^67^ were employed to calculate the electron repulsion integrals. During the DFT-level optimization, only the positions of the dangling H atoms of the cluster models were kept fixed to their original positions, while the atoms of the zeolite framework and extraframework ensemble were fully relaxed.

The energies of the lowest-lying structures after the optimization at the DFT level of theory were further employed for ab initio thermodynamic analysis. Ab initio thermodynamic analysis was conducted to account for the temperature and pressure effects in the presence of water on the stability of the extraframework species. The relative energies were computed with the reference to water, pure Ca-ZSM-5, H-ZSM-5, and bulk β-Ga_2_O_3_ structures, which are provided in the Supporting Information. The equilibria between species were established to have the following general form for the formation of the Ca–Ga and Ga-only structures
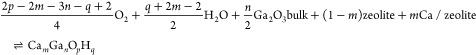
6where Ca_*m*_Ga_*n*_O_*p*_H_*q*_ is the total electronic energy of one of the global minima, zeolite is the energy of the H-form of the ZSM-5 structure with two framework Al atoms, and Ca/zeolite is the total energy of the ZSM-5 structure with two framework Al atoms compensated by an exchangeable Ca^2+^ cation. The O_2_, H_2_O, and Ga_2_O_3_bulk are the total energies of gaseous O_2_, H_2_O, and bulk Ga_2_O_3_, respectively. The vibrational and pressure–volume contributions of solids were neglected and their Gibbs free energies were approximated as their respective electronic energies. The chemical potentials of gaseous water and oxygen species were calculated with respect to the reference state at 0 K and 1 bar using tabulated thermodynamic tables.^68^

The reaction Gibbs free energy Δ*G*(*T*,*p*) equals to
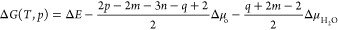
7where the reaction energy Δ*E* and the chemical potential of water μ_H_2_*O*_(*T*,*p*) at an arbitrary temperature *T* and pressure *p* are defined as follows
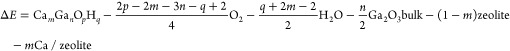
8
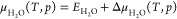
9

The expression for the chemical potential change includes the temperature- and pressure-dependent free energy contributions as follows
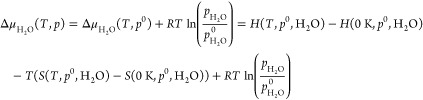
10

Ethane dehydrogenation was chosen as the representative model reaction to computationally assess the dehydrogenation reactivity of the extraframework LAS. The Lewis acidic cationic clusters were stabilized within the periodic ZSM-5 model with the optimized unit cell lattice parameters of *a* = 20.2 Å, *b* = 20.0 Å, *c* = 13.4 Å, and α = β = γ = 90°, which were kept fixed throughout the calculations. Periodic density functional theory (DFT) calculations were carried out at the PBE-D3(BJ) level of theory^69,70^ using the Vienna Ab Initio Simulation Package (VASP 5.3.5).^71,72^ The plane wave basis set with an energy cutoff of 450 eV and the projector augmented wave (PAW)^73^ method were used. Brillouin zone sampling was restricted to the Γ point. The convergence was considered to be reached when the forces acting on each atom were below 0.05 eV Å^–1^. The minimum reaction energy path and the transition states search were performed by employing the nudged-elastic band (CI-NEB) method.^74^ The geometry corresponding to the maximum energy structure along the reaction path was further optimized via a quasi-Newton algorithm, where only the relevant atoms of the extraframework species were relaxed. The finite difference method was used to calculate the vibrational frequencies (0.02 Å atomic displacements). The energy barrier for the β-H elimination was disregarded on the grounds of earlier reports that indicate that this elementary step depends only slightly on the coordination environment of the Ga atom^75^ and therefore cannot give rise to the diverging dehydrogenation activity.

## 3 Results

### 3.1 Catalyst Characterization

The results present in Table S1 show that the elemental composition of each sample is well in line with calculations. The obtained XRD patterns shown in Figure S1a and unit cell parameters of bimetallic samples in Table S2 confirm that main diffractions corresponding to the MFI-type zeolite framework were preserved, while the crystallinity is slightly decreased for Ca,Ga-modified catalysts except for Ca(0.05)Ga(2) after metal addition and following thermal treatments (reduction, oxidation, and calcination). The pore structure of each sample is assessed by N_2_ physisorption tests, and the results given in Figure S1(b) show that the micropore volume was slightly reduced from 0.15 to 0.14 cm^3^/g, while the BET surface area decreased from ∼420 to 379 m^2^/g for H-ZSM-5 and Ga(2), respectively. Due to the small loading of Ca (0.02–0.5 wt %) on Ga(2), the changes of pore volumes and BET surface area related to Ca addition are negligible as shown in Figure S1b.

**Figure 1:**
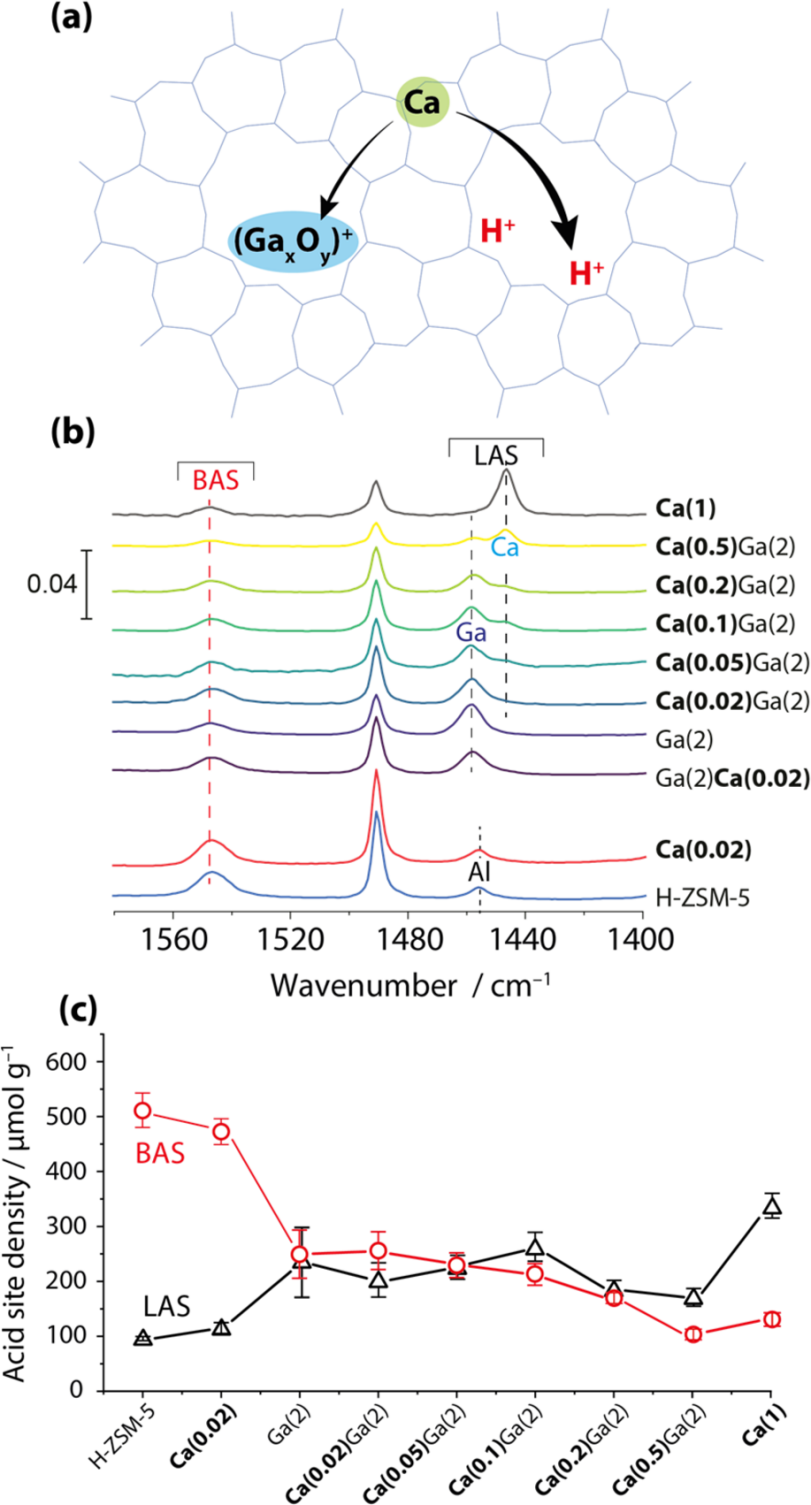
Acidity characterization of mono- and bimetallic [Ca,Ga]/H-ZSM-5 catalysts by FTIR measurements using pyridine as probe. (a) Schematic illustration of Ca doping on Ga oxide species and BAS in the H-ZSM-5 zeolite framework, (b) FTIR spectra with pyridine adsorption on zeolites with different metal loadings, and (c) acid site density of BAS and LAS determined by integrating bands at 1550 and 1460–1440 cm^–1^. The spectra with pyridine adsorption were obtained at 160 °C. Error bars represent the standard deviations of the quantitative analysis results from at least two measurements for each sample.

Figure 1 shows the characterization of acid sites of bimetallic catalysts obtained from FTIR spectroscopy measurements with pyridine as the probe molecule. The IR spectra feature the characteristic bands of pyridine adsorbed on BAS and LAS (Figure 1b). A band at 1547 cm^–1^ observed for all catalysts corresponds to pyridine interacting with the BAS.^45^ Pyridine interaction with LAS gives rise to bands in the 1458–1446 cm^–1^ range.^27,76−78^ The band at 1455 cm^–1^ observed for H-ZSM-5 and Ca(0.02) is assigned to the extraframework Al species.^78,79^ At Ca loading >0.1 wt %, a band at 1446 cm^–1^ appears which can be attributed to the formation of Ca-LAS at the ion-exchange sites (BAS). Ga-promoted catalysts feature a characteristic band at 1458 cm^–1^ due to the formation of Ga-LAS.^80^ Previous research reveals that these observed IR band shifts in the 1458–1446 cm^–1^ range can be attributed to the formation of LAS with different strengths and therefore different adsorption energies.^78^ Therefore, the Ga-LAS featuring the FTIR band at 1458 cm^–1^ possesses the strongest acidity in comparison with extraframework Al (1455 cm^–1^) and Ca-LAS (1446 cm^–1^).

The quantitative analysis (Figure 1c) of these bands shows that the addition of 2 wt % Ga introduces ca. 150 μmol g^–1^ Ga-LAS at the expense of 230 μmol g^–1^ BAS. 0.02 wt % Ca addition to the parent H-ZSM-5 shows a substantial decrease (∼20 μmol g^–1^) in BAS concentration. 1 wt % Ca addition significantly reduces the BAS concentration from 512 to 130 μmol g^–1^ for H-ZSM-5 and Ca(1), respectively. This is ascribed to the high affinity of Ca to accommodate at the ion-exchange sites (BAS, Figure 1a), resulting in a BAS IR band of lower intensity and a Ca-LAS band of higher intensity [compare Ca(1) with H-ZSM-5 in Figure 1b]. For the same reason, the addition of larger amounts of Ca (>0.05 wt %) on Ga(2) reduces the BAS concentration, while the concentration of newly formed Ca-LAS increases, as shown in Figure 1b,c. Up to 0.05 wt % Ca addition, no visible changes in BAS concentration for Ga(2) can be noted. However, the concentration of Ga-LAS decreases with a simultaneous Ca-LAS increase upon Ca addition (Figure 1b). This is interpreted as that at these conditions Ca interacts with Ga extraframework species rather than exchanges with protons of BAS (Figure 1c).

The additional evidence for the change of the acidity after Ca addition comes from the results of FTIR spectroscopy measurements using CD_3_CN as a probe. As shown in Figure S2, the spectrum of the parent H-ZSM-5 features the prominent band at 2300 cm^–1^ with two weak bands at 2285 and 2265 cm^–1^ due to CD_3_CN adsorbed on BAS, silanol (SiOH) groups, and physisorbed CD_3_CN, respectively.^81,82^ The band at 2320 cm^–1^ is attributed to Lewis acidic EFAl sites. For Ga(2), the intensity of the BAS band at 2300 cm^–1^ decreases, while two new bands at 2316 and 2326 cm^–1^ appear in the spectrum due to the formation of new Ga LAS sites with different strengths formed upon the exchange of the parent BAS in the zeolite. The addition of 0.02 wt % Ca gives rise to further substantial changes of the IR spectrum of adsorbed CD_3_CN. The maximum of the band due to Ga-LAS shifts from 2326 to 2323 cm^–1^ and decreases in intensity (relative to BAS), suggesting the weaker Lewis acidity of the respective sites formed after the introduction of Ca.

### 3.2 MTA Activity Tests

**Figure 2:**
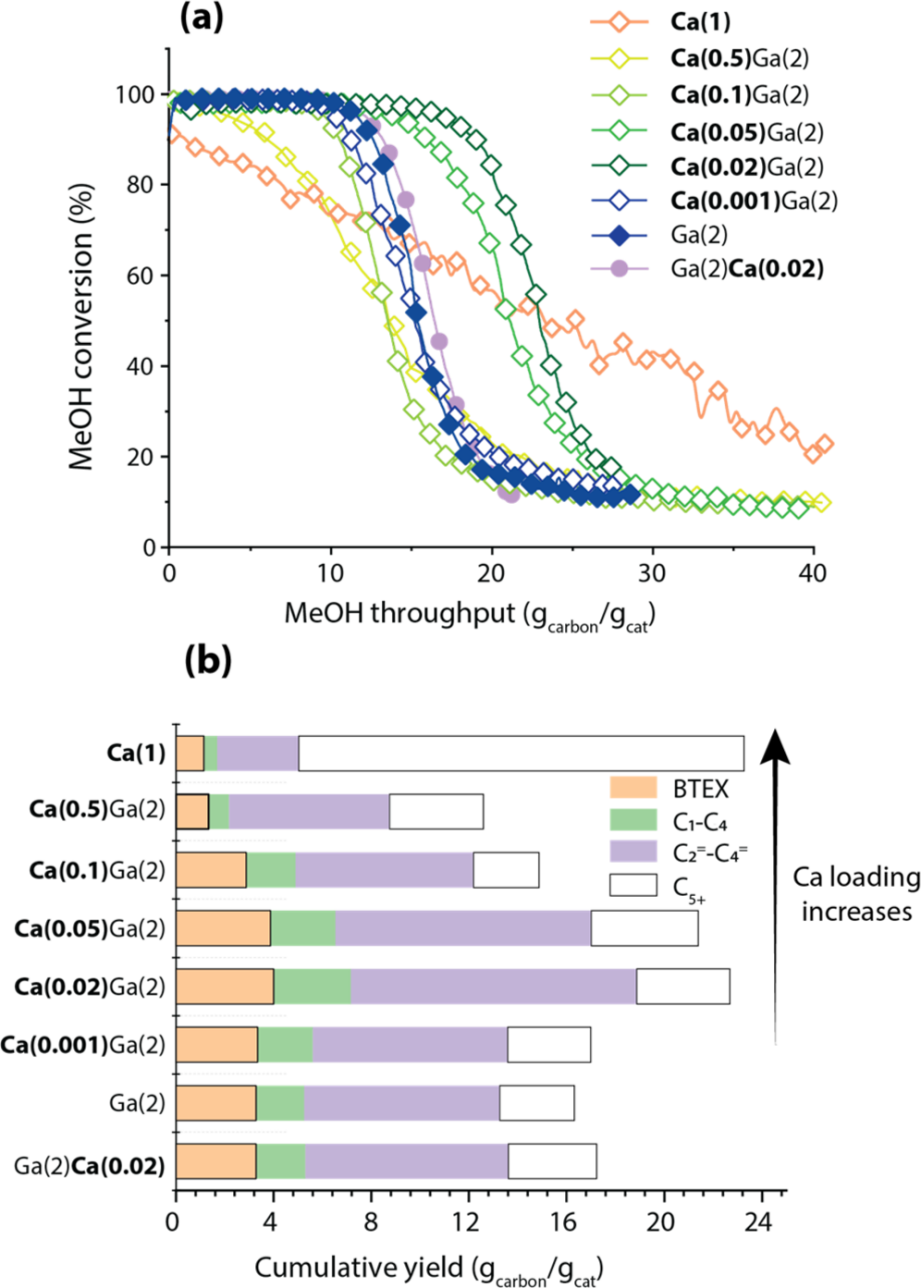
Summary of the catalytic results of MTA over Ca,Ga-modified catalysts: (a) MeOH conversion as a function of MeOH conversion per gram of catalyst; (b) Integral yields of the main groups of MTA products before MeOH conversion drops below 20%. MTA reaction conditions: *T* = 450 °C, *m*_cat_ = 40 mg (150–212 μm), *P*_reactor_ = 1 bar, WHSV = 5.3 g_MeOH_ g_cat_^–1^ h^–1^, and carrier gas N_2_ = 50 mL min^–1^. C_5+_: aliphatics with the carbon number higher than 4; C_1_–C_4_: C_1_ to C_4_ alkanes; and C_2_^=^–C_4_^=^: C_2_ to C_4_ olefins. The full picture of product distribution as a function of MeOH throughput is given in Figure S4.

Ga-modified zeolites are well-known to catalyze the dehydrogenation of various substrates such as alkanes into olefins and aromatics.^26,27,37,83,84^ Accordingly, in the MTA process, MeOH is first converted into the primary hydrocarbons,^85,86^ after which the aromatization proceeds via the dehydrogenation reaction path over Ga-LAS^27,37,38^ with the danger of further condensation to polyaromatics and deactivation of the catalyst. To test the stability of the as-prepared catalysts under industrially steady-state conditions, the MTA was carried out under the same WHSV (5.3 g_MeOH_ g_cat_^–1^ h^–1^) when MeOH is fully converted into primary hydrocarbons (MeOH conversion is 100%). Along with MTA reactions proceeding, MeOH conversion drops quickly after different times on stream and finally stabilizes at ca. 10% (Figures S3 and S4). Accordingly, the product cumulative yield was calculated by integrating the carbon yield during the entire lifetime (MeOH conversion from 100 to 20%). The main results of the MTA conversion over monometallic Ga-, Ca-as well as bimetallic Ca,Ga-modified H-ZSM-5 zeolite catalysts are presented in Figures 2, S3, and 4.

Catalytic tests for Ga-modified H-ZSM-5 catalysts with varying Ga loadings (Figure S3) show that the carbon yield of BTEX increased from 13% (at cumulative MeOH throughput of 5 g_carbon_/g_cat_) for parent H-ZSM-5 to 18% and then to 24% for Ga(1) and Ga(2) samples. A further increase in Ga content, however, had a rather negative effect on BTEX selectivity. This can be attributed to a previously reported agglomeration of Ga species and a decreased Ga dispersion at high Ga loadings.^37^ Therefore, the Ga(2) catalyst ensures the highest BTEX selectivity and was chosen as the starting material for Ca doping.

Figures 2 and S4 summarize the MTA results obtained for bimetallic Ca,Ga-modified H-ZSM-5 samples with 2 wt % Ga and Ca loading ranging from 0.02 to 0.5 wt %. The results indicate that upon the addition of only 0.02 wt % Ca to Ga(2), the total MeOH throughput increases from 16 to 23 g_carbon_/g_cat_ for Ga(2) and Ca(0.02)Ga(2), respectively. Accordingly, the integral yield of BTEX increases from 3 to 4 g_carbon_/g_cat_ and that of light olefins increased from 8 to 12 g_carbon_/g_cat_ for Ga(2) and Ca(0.02)Ga(2), demonstrating a strong effect of low Ca loadings on the catalytic performance of Ga-modified zeolites. The lifetime extension impact gradually diminishes with a further increase in Ca loading to 0.05 and 0.1 wt %. Upon 0.5 wt % addition to Ga(2), the MeOH conversion rapidly drops, resulting in a total MeOH throughput and integral BTEX yield of only 12 and 2 g_carbon_/g_cat_, respectively. Unlike Ca,Ga-modified catalysts, Ca(1) exhibits the incomplete MeOH conversion immediately followed by a slow deactivation. As a result, the total MeOH throughput is 23 g_carbon_/g_cat_, in which more than 18 g_carbon_/g_cat_ is converted into bulky C_5+_ aliphatics. This can be attributed to the limited cracking activity of Ca(1) at a relatively low temperature of 450 °C used in this study, giving rise to the accumulation of the oligomeric species in the zeolite pores and, consequently, catalyst deactivation (Figure 2a).^43,87^

To ensure that the observed changes in MTA performance are not related to the preparation procedure of bimetallic samples, a Ga(2) catalyst containing trace amounts of Ca (∼0.001%) was prepared, following the same procedure. For this sample, no changes in performance as compared to pure Ga(2) catalyst were observed (Figure 2). Moreover, the same reaction performance was also observed for Ga(2)Ca(0.02), the sample prepared following a similar protocol but with metal addition in the reversed order.

**Figure 3:**
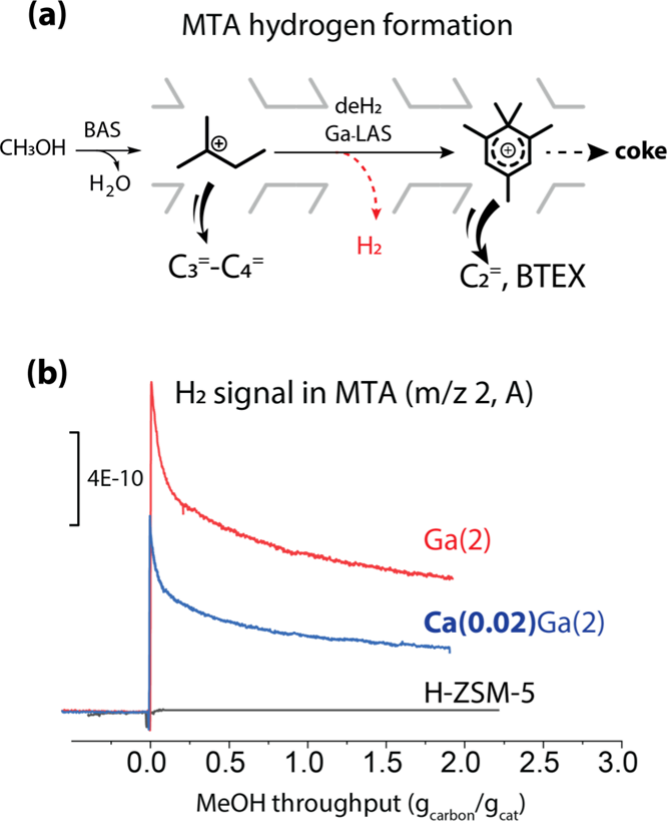
Schematic illustration of MeOH aromatization with hydrogen formation over Ga-LAS (a) and hydrogen formation along MeOH throughput at the initial stage of MTA tests over parent H-ZSM-5 and Ca,Ga-modified catalysts (b).

To further elucidate the catalytic impact of 0.02 wt % of Ca on Ga(2) in the MeOH aromatization process, hydrogen formation is used as a descriptor of dehydrogenation reaction (Figure 3a).^27^ As shown in Figure 3b, during MTA tests, a negligible amount of hydrogen is formed over the parent H-ZSM-5. In turn, the lower hydrogen formation is observed for Ca(0.02)Ga(2) compared to Ga(2), suggesting that the direct dehydrogenation is suppressed in the presence of Ca. Combining all these results, we propose that the increased MeOH throughput and BTEX production over Ca(0.02)Ga(2) are related to the small amount of Ca affecting the intra-zeolite Ga species and their (Lewis) acidic properties, moderating the dehydrogenation activity.

We have carried additional characterization of selected catalysts using the 3-MP cracking as the probe (Figure S5). Compared to H-ZSM-5, 3-MP cracking conversion and H_2_ formation simultaneously increase over Ga-modified catalysts, evidencing the promoted dehydrogenation over Ga-LAS (top route in Figure S5a). Upon 0.02–0.5 wt % Ca addition to Ga(2), the selectivity to methane and ethane significantly decreases, indicating the suppression of the monomolecular cracking (mid route in Figure S5b). Simultaneously, the H_2_ formation also decreases slightly in line with the proposed moderated dehydrogenation activity of Ca(0.02)Ga(2). The higher 3-MP conversion over Ca(0.02)Ga(2) than over Ga(2) is therefore attributed to the increased rate of the bimolecular cracking, via H-transfer reactions (bottom route in Figure S5a). At 0.5 wt % of Ca loading, the formation of methane, ethane, and H_2_ is suppressed, suggesting the prevalence of the bimolecular cracking path. These results point to the higher rate of the H-transfer reactions over Ca(0.02)Ga(2) due to the moderated dehydrogenation activity. Increased Ca loading, however, reduces the rates of cracking and dehydrogenation caused by BAS exchanged by Ca. In the presence of water, both 3-MP conversion and H_2_ formation decrease over selected catalysts, implying the decreasing dehydrogenation activity with the degree of hydration of the Ca,Ga clusters.

Overall, the bimetallic Ca(0.02)Ga(2) catalyst demonstrates an improved stability and increased yield of BTEX in MTA in comparison with Ga(2). On the contrary, the catalysts in which Ca loadings are higher than 0.05 wt % show decreased stability because Ca exchanges with protons of BAS forming Ca-LAS.^43^ For Ca(1), the incomplete MeOH conversion followed by a slow deactivation is observed. Following the previous investigations in the group, the presence of Ca-LAS next to BAS decreases the stability and growth rate of aromatic hydrocarbon pool intermediates,^43,88^ which also explains the highest yield of C_5+_ over Ca(1) (Figure 2b). At 450 °C which is below the reported >500 °C for Ca-modified ZSM-5 in MTO in refs (43)(88), the cracking of these C_5+_ aliphatics is also limited over the remaining BAS in Ca(1) eventually causing the gradual deactivation.

On the basis of these data at small loadings (Ca < 0.05 wt %), we propose that Ca first interacts with extraframework Ga species and the synergy of Ca and Ga moderates the dehydrogenation rate. This leads to the lower hydrogen formation and eventually reduces the deactivation rate in MTA. At higher Ca loadings (>0.05 wt %) on Ga(2), the dehydrogenation–aromatization rate is further suppressed. However, more Ca atoms inevitably interact with BAS, forming Ca-LAS (Figure 1b,c). Newly formed Ca-LAS forces the MeOH transformations into C_5+_ aliphatics rather than olefins (cracking) or BTEX (dehydrogenation), causing the fast deactivation of Ca(1) or Ca(0.5)Ga(2) as presented in Figures 2 and S4. Note that the catalyst preparation approach employed in this study cannot decouple the formation of CaGa binuclear species and Ca exchanging with protons of BAS. Our data suggest that when targeting higher Ca loadings, the latter process becomes dominant, resulting in the replacement of BAS with exchangeable Ca^2+^ ions and, accordingly, the overall deterioration of the catalyst performance.

### 3.3 Computational Results

In an attempt to provide a molecular proposal for the observed reactivity changes upon Ca modification of Ga/H-ZSM-5, model DFT calculations were carried out. Following the hypothesis on Ca-mediated reactivity changes in extraframework Ga sites, a fully automated analysis of the interaction modes^51^ between Ca^2+^ and representative binuclear Ga_2_O_*x*_H_*y*_ moiety was carried out. The calculations were expanded into the *operando* regime through the ab initio thermodynamics (aiTD) analysis to find out the extraframework complexes potentially formed under the MTA conditions.^89^

Following on earlier works on Ga-modified H-ZSM-5 materials, we have considered the model of the active site consisting of a binuclear Ga cluster stabilized by two negatively charged aluminum, incorporated in the MFI framework with a different environment such as alpha, beta, and gamma sites.^90−92^ The alpha and beta sites are the six-membered rings along the straight channel, whereas the gamma site is the eight-membered ring on the wall of the sinusoidal channel.^93^ The effect of Ca addition was studied by introducing one Ca^2+^ cation. The overall charge neutrality of the pure Ga or CaGa bimetallic species was achieved by introducing the O^2–^ and OH^–^ ligands, whose quantity was varied to represent different water contents. This resulted in the structures containing a water content of 0–5 H_2_O molecules, giving six stoichiometries for pure Ga and six for CaGa structures.

To find the global minima structures corresponding to these stoichiometries, a genetic algorithm optimization process was carried out, with the electronic structure evaluation calculated by an accelerated *x*TB semi-empirical method.^50,54^ As the exhaustive computational search of the 96 T periodic atom-system is currently prohibitively demanding, the cluster models representing the Ga pure and CaGa bimetallic active sites confined in the sites of the ZSM-5 were utilized.^51^ The outcome of each genetic algorithm procedure was 20 lowest-lying configurations of the corresponding stoichiometry, with indicated structural diversity, whose geometries were further refined at the PBE-D3(BJ) level of theory with Gaussian DZVP-MOLOPT-GTH basis set as implemented in CP2K 6.1.^55,56,58,60−66^ The stability of the lowest high-level refined structures of each stoichiometry was further assessed at experimentally relevant conditions employing aiTD (eqs6−10).^94^ The cluster models of the global minima for all stoichiometries are shown in Figures S6–S9.

**Figure 4:**
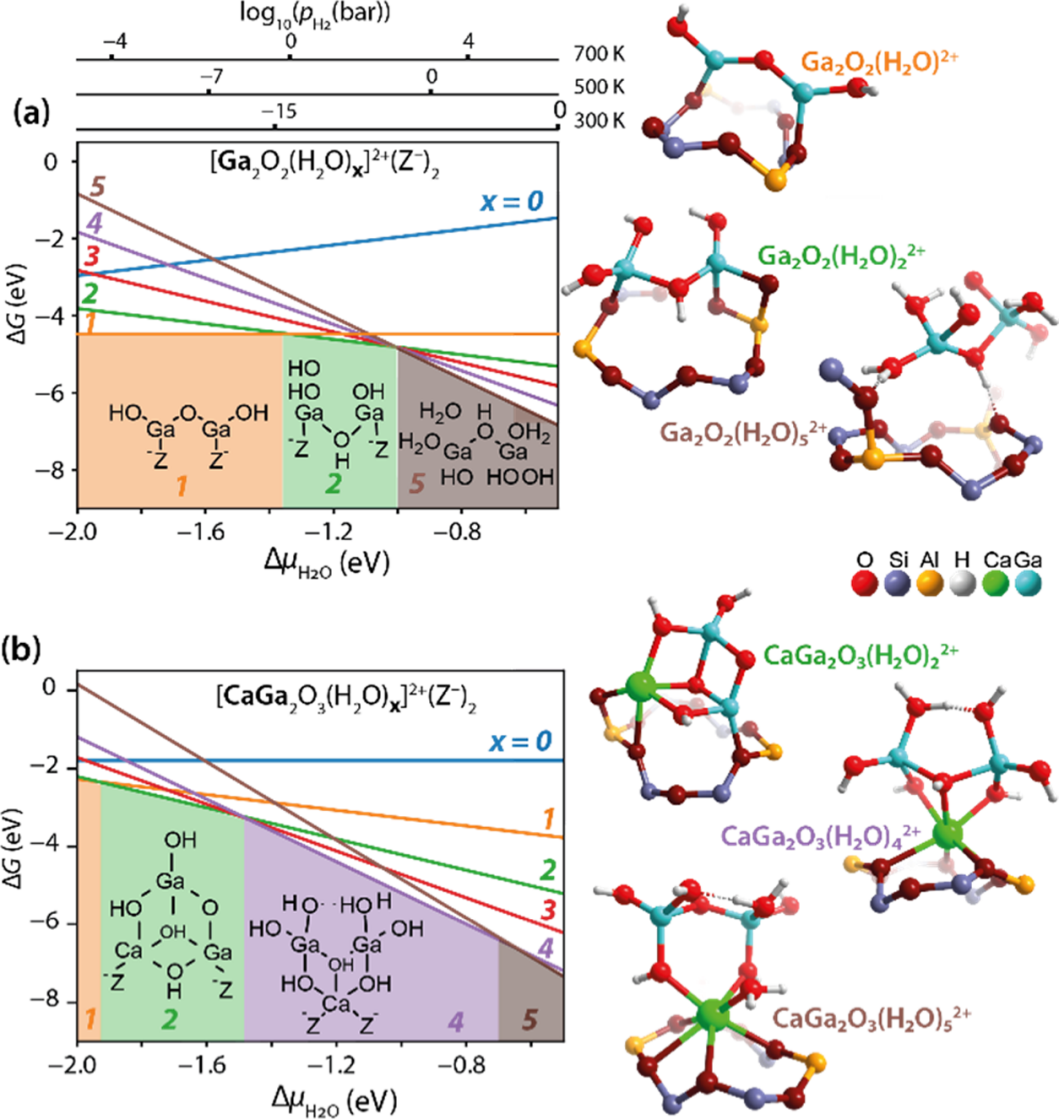
Stability and geometries of cationic extraframework species in ZSM-5 zeolite with different water contents (up to five water molecules). (a) aiTD analysis on the pure Ga complexes. The geometries of the most stable configurations among the following stoichiometries: Ga_2_O_2_(H_2_O)^2+^, Ga_2_O_2_(H_2_O)_2_^2+^, and Ga_2_O_2_(H_2_O)_5_^2+^ are displayed. The geometry of Ga_2_O_2_(H_2_O)_5_^2+^ illustrates the detaching from the framework. (b) aiTD analysis on the bimetallic CaGa complexes. The geometries of the most stable configurations among the following stoichiometries: CaGa_2_O_3_(H_2_O)_2_^2+^, CaGa_2_O_3_(H_2_O)_4_^2+^, and CaGa_2_O_3_(H_2_O)_5_^2+^ are displayed.

The optimized geometries of the most stable pure Ga and bimetallic CaGa configurations within each stoichiometry and their relative stabilities as a function of reaction conditions are shown in Figure 4. The comparison of the optimized geometries reveals that at all hydration levels, Ga ions in both Ga pure and Ga,Ca extraframework clusters tend to adapt a distorted tetrahedral coordination environment. The only observed exception is the trigonal bipyramidal coordination formed around one of the gallium centers in the Ga_2_O_2_(H_2_O)_4_^2+^ model (Figure S7a). The coordination of the Ca ions in the bimetallic clusters depends more strongly on the water content. In the presence of 1 or 2 water molecules (Figure S8b,c), the coordination of the Ca^2+^ center in the Ga,Ca clusters is best described as the square pyramidal, whereas at a higher solvation level (with 3, 4, or 5 added H_2_O molecules), distorted pentagonal bipyramidal or octahedral coordinations of the Ca centers are realized in the extraframework clusters (Figures S8d and S9a,d).

Accordingly, at the low values of the water chemical potential (−2.0 eV < Δμ < −1.2 eV), pure Ga-oxo species tend to coordinate one or two water molecules, whereas bimetallic CaGa species favor the hydration with up to four water molecules. At intermediate and high values of Δμ (>−1.2 eV), pure Ga species can coexist with configurations having the various degrees of hydration (Δμ ∼ −1 eV) and eventually get hydrolyzed (Δμ > −1 eV). At high water chemical potential, the coordination of five water molecules to the pure Ga species leads to the hydrolysis of Si–O–Ga bonds, resulting in the detachment of the species from the framework, as in structure Ga_2_O_2_(H_2_O)_5_^2+^ in Figure 4a. However, the bimetallic cations remain effectively attached to the cation site at all Δμ through coordinating Ca to the framework Al sites. This effect is illustrated with the most stable bimetallic CaGa_2_O_3_(H_2_O)_4_^2+^ and CaGa_2_O_3_(H_2_O)_5_^2+^ configurations (Figure 4b) suggesting that Ca acts as an anchor, preventing the highly hydrated extraframework species from washing away from the cation site and agglomerate. The aiTD diagrams indicate that Ca addition stabilizes the bimetallic species with a higher degree of hydration (containing more water molecules) rather than pure Ga configurations at the same conditions.

Furthermore, the dehydrogenation activity of the pure Ga and CaGa complexes, stabilized at intermediate water chemical potentials (mimicking the MTA conditions), was assessed by using ethane dehydrogenation as a model test reaction.^75,91,95−97^ Specifically, the reactivity of Ga_2_O_2_(H_2_O)^2+^, Ga_2_O_2_(H_2_O)_2_^2+^, and their Ca-containing counterparts CaGa_2_O_3_(H_2_O)_2_^2+^ and CaGa_2_O_3_(H_2_O)_4_^2+^ were computationally assessed. Ethane dehydrogenation proceeds via the following elementary steps, namely, the heterolytic C–H-bond cleavage, β-elimination, and H_2_ recombination (Figures S10 and 11). The reaction energies and activation barriers of the respective steps are summarized in Table S3.

DFT calculations indicate that the Lewis acidity and the dehydrogenation activity of the intrazeolite clusters decrease with the increase in hydration levels, which are more favored for the bimetallic Ca,Ga clusters (Figures S10 and 11). Under the conditions relevant for the MTA reaction (Δμ_H_2_O_ > −1.2 eV, Figure 4), the dominant bimetallic CaGa_2_O_3_(H_2_O)_4_^2+^ clusters exhibit computed barriers for the C–H activation and H_2_ recombination that are 32 and 10 kJ/mol, respectively, higher compared to its Ga-only counterpart Ga_2_O_2_(H_2_O)_2_^2+^. The current reactivity assessment specifically focused on the impact of the change of the properties of the Lewis acidic Ga center on the dehydrogenation activity. We anticipate that similar to other intrazeolite active complexes, the reactivity of the Ga-containing multinuclear clusters depends on a wide variety of secondary effects such as the presence of multiple reaction channels,^98^ active site dynamics,^99^ and the variation of the local zeolite environment.^100^ The detailed investigation of these factors is beyond the scope of the present study and is a focus of the ongoing computational efforts in our group.

Therefore, we propose that the addition of Ca allows us to sustain the catalytic CaGa complexes in a more hydrated state during the MTA reaction. The higher degree of hydration for the CaGa system results in a higher barrier for the C–H bond cleavage, moderating thus effectively the rate of the dehydrogenation paths of the MTA reaction.

## 4 Conclusions

MTA over Ga-modified zeolites offers a sustainable route for the production of important commodities such as benzene, ethylbenzene, toluene, and xylenes. The increase in the selectivity toward the aromatics is accompanied by issues of enhanced coke deposition and subsequent early deactivation of the catalyst. The addition of minute amounts of Ca (0.02 wt %) prolongs the lifetime of the catalyst while maintaining a high selectivity toward aromatics. The Ca(0.02)Ga(2) converted 43% more MeOH and gave 33% higher yield of BTEX than Ga/H-ZSM-5 before the catalyst was fully deactivated. Higher Ca loadings (>0.05 wt %) not only give rise to Ca-LAS formation but also diminish the impact of lifetime extension in MTA.

The mechanistic basis of the catalytic impact of Ca in the MTA process depending on Ca loading is still unclear. Based on the MTA performance and IR spectroscopy analysis, we propose that the minute addition of Ca to Ga-modified zeolites ensures the formation of CaGa extraframework clusters, reducing Ga-LAS, before Ca starts exchanging with protons of BAS. The interaction of Ca and Ga results in a moderated dehydrogenation rate evidenced by the lower hydrogen formation over Ca(0.02)Ga(2) than Ga(2) in the MeOH aromatization process. The computational modeling suggests that the Ca^2+^ cation added to the Ga extraframework structure allows it to accommodate more water molecules exhibiting a lower Lewis acidity and a higher stability under water-containing conditions. Accordingly, the higher C–H bond activation energy barrier over CaGa clusters leads to reduced dehydrogenation activity and a slower deactivation process.

The targeted modification of Ga extraframework species with small quantities of Ca is demonstrated as a promising approach for the further optimization and practical implementation of the MTA process.

## Supporting Information Available

Metal contents determined by ICP analysis; XRD patterns; unit-cell lattice parameters and crystallinity results of all catalysts; FTIR spectra; MeOH conversion and carbon yields of BTEX and MTA; DFT-optimized structures of intermediates and transition states; global minima for all stoichiometries; DFT-computed reaction energy diagrams; computed energetics of the C−H-bond activation, β-elimination, and H_2_ recombination steps (ZIP)
