# Supplementary material for: High Stability of Methanol to Aromatic Conversion over Bimetallic Ca,Ga-Modified ZSM-5
Source: ACS Catal. 2022 Feb 23;12(5):3189–200. doi: 10.1021/acscatal.1c05481 (PMC8902757; doi:10.1021/acscatal.1c05481)
Supplement: Supplementary file 1 — cs1c05481_si_001.zip [file cs1c05481_si_001.zip › cs1c05481_si_001_REV.pdf]

SUPPORTING INFORMATION FOR

High stability of methanol to aromatic conversion over bimetallic Ca,Ga-modified ZSM-5

Chuncheng Liu,[a,c] Evgeny A. Uslamin,[a] Elena Khramenkova,[a] Enrico Sireci,[a] Lucas (T.L.J.) Ouwehand,[a] Swapna Ganapathy,[b] Freek Kapteijn[c]* and Evgeny A. Pidko[a]*

[a] Inorganic Systems Engineering, Department of Chemical Engineering
Delft University of Technology
Van der Maasweg 9, 2629 HZ Delft, The Netherlands

[b] Radiation Science and Technology Department
Delft University of Technology
Mekelweg 15, 2629 JB Delft, The Netherlands

[c] Catalysis Engineering, Department of Chemical Engineering
Delft University of Technology
Van der Maasweg 9, 2629 HZ Delft, The Netherlands

*: Evgeny A. Pidko, e.a.pidko@tudelft.nl; Freek Kapteijn, F.Kapteijn@tudelft.nl

KEYWORDS: methanol-to-aromatics, bimetallic catalyst, dehydrogenation, global optimization, catalyst deactivation

S1. Supplementary Experimental Results

Table S1. Prepared samples with metal contents determined by ICP analysis.

	Si/Al molar ratio	Ca(wt%)	Ga(wt%)	Al(wt%)	Ca/Al molar ratio	Ga/Al molar ratio
H-ZSM-5	25	-	-	1.60	-	-
Ga(2)	25	-	2.15	1.57	-	0.530
Ca(1)	25	0.90	-	1.60	0.380	-
Ca(0.02)Ga(2)	25	0.03	2.18	1.57	0.009	0.538
Ca(0.05)Ga(2)	25	0.04	2.05	1.58	0.017	0.502
Ca(0.1)Ga(2)	25	0.07	2.22	1.60	0.030	0.537
Ca(0.5)Ga(2)	25	0.62	2.15	1.62	0.258	0.514
Ga(2)Ca(0.02)	25	0.02	2.07	1.61	0.008	0.496

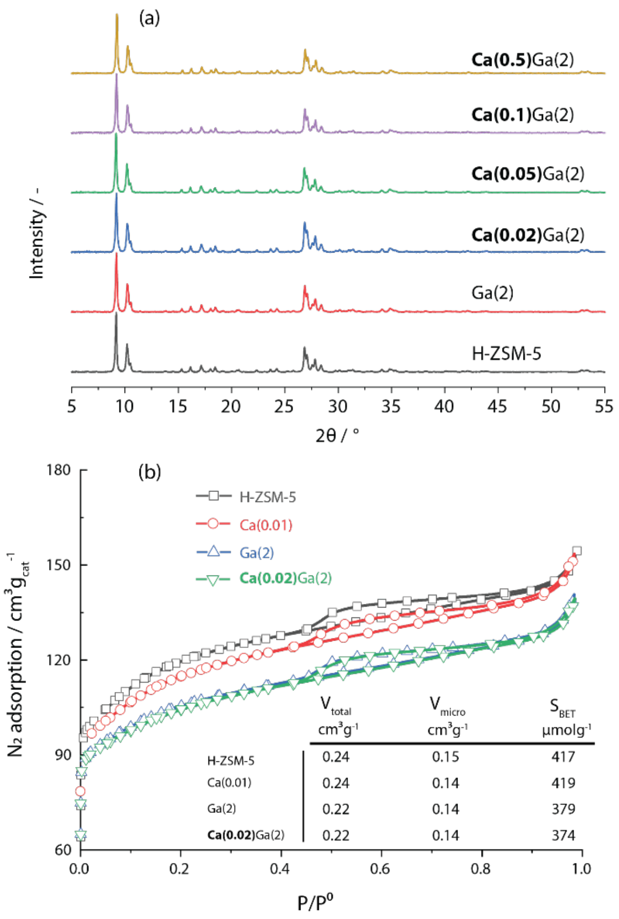

Figure S1. X-ray diffraction data comparing patterns for the investigated samples (a), and N_2 adsorption-desorption isotherms (b).

Table S2. Unit-cell lattice parameters and crystallinity results of all catalysts under study.

	Crystallinity (%)	*a* (Å)	*b* (Å)	*c* (Å)	Crystal size (Å)
H-ZSM-5	97.0	20.090	19.882	13.391	644
Ga(2)	96.5	20.108	19.908	13.391	606
Ca(0.02)Ga(2)	93.2	20.094	19.905	13.393	601
Ca(0.05)Ga(2)	97.3	20.084	19.901	13.390	611
Ca(0.1)Ga(2)	92.1	20.094	19.893	13.386	638
Ca(0.5)Ga(2)	93.7	20.164	19.902	13.369	588

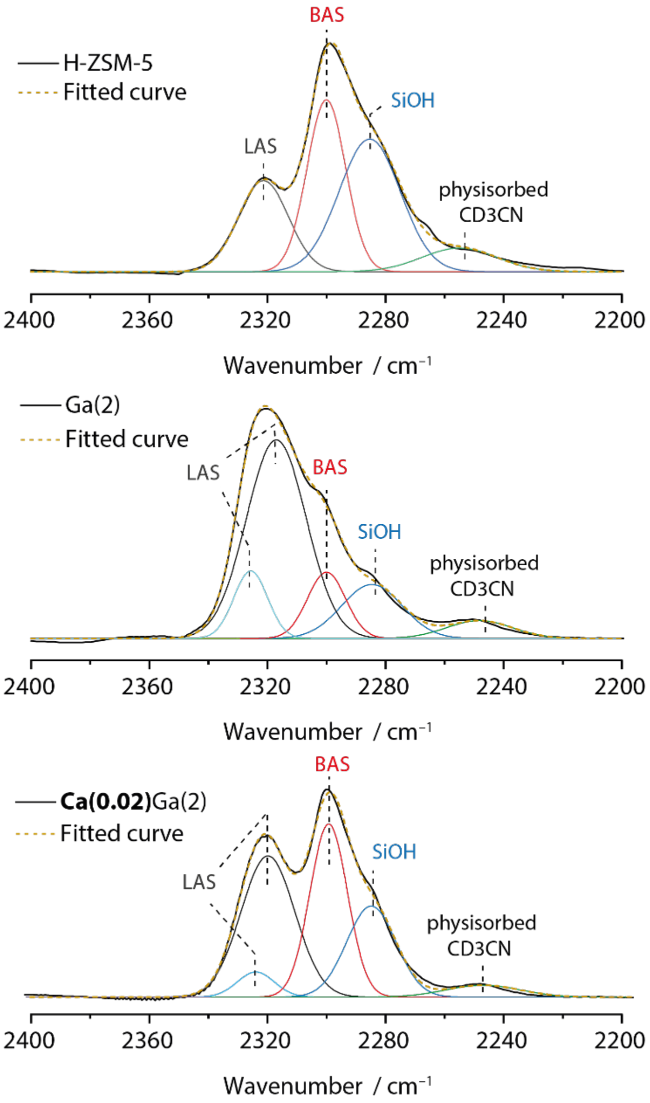

Figure S2. FTIR spectra of acetonitrile-d3 (CD_3CN) adsorbed on H-ZSM-5, Ga(2), and Ca(0.02)(Ga2). Spectra were collected at room temperature by periodically dosing a little amount of CD_3CN vapour till saturation (~ 2 mbar). The curve fitting was performed using Voigt function.

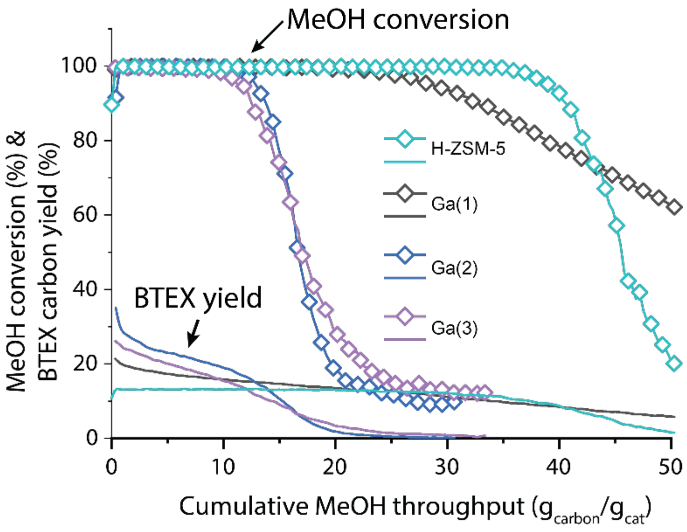

Figure S3. MeOH conversion and carbon yields of BTEX as a function of cumulative MeOH throughput over parent H-ZSM-5 and Ga-modified ZSM-5 catalysts. Reaction conditions: $T = 450$ °C, $m_{cat} = 40$ mg (150–212 μm), $P_{reactor} = 1$ bar, $WHSV = 5.3$ $g_{MeOH}g_{cat}^{-1}h^{-1}$, carrier gas $N_2 = 50$ mL min^{-1}.

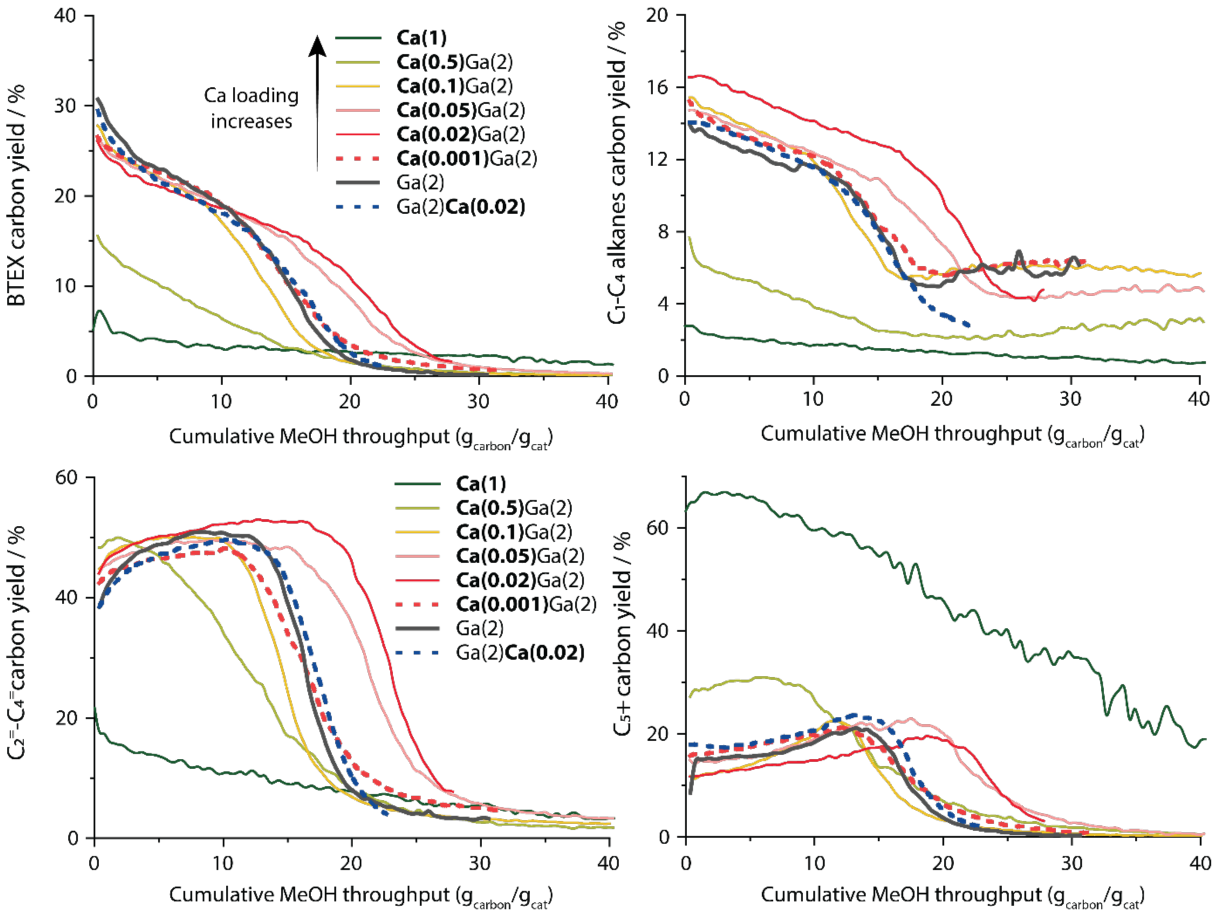

Figure S4. Carbon yields of MTA products (BTEX, C_1-C_4, $C_2^=$-$C_4^=$ and others) as a function of cumulative MeOH throughput over Ga,Ca-modified ZSM-5 catalysts. Reaction conditions: T = 450 °C, m_{cat} = 40 mg (150–212 μm), $P_{reactor}$ = 1 bar, $WHSV$ = 5.3 $g_{MeOH}g_{cat}^{-1}h^{-1}$, carrier gas N_2 = 50 mL min^{-1}.

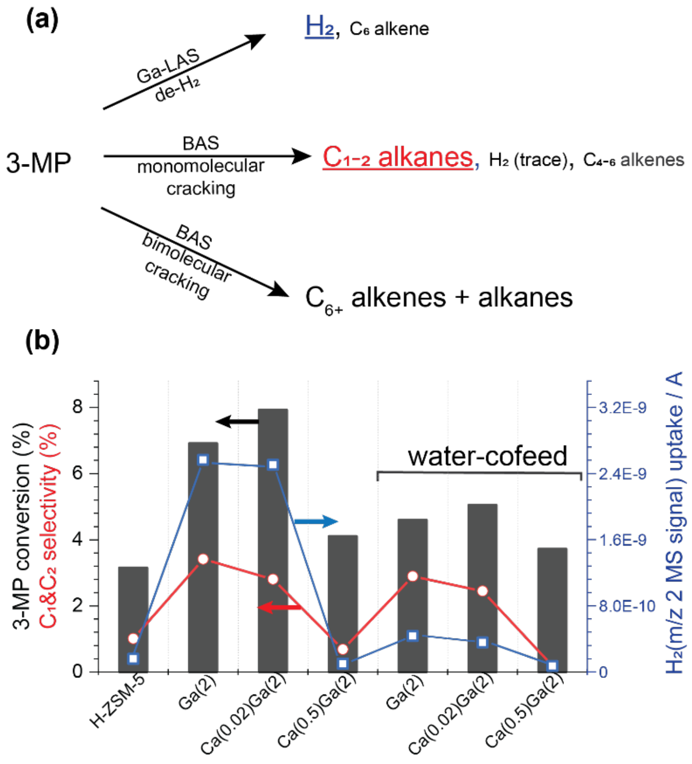

Figure S5. Reaction mechanisms of 3-methylpentane (3-MP) cracking over Ga modified catalysts **(a)**; 3-MP cracking conversion and light cracking products over parent H-ZSM-5 and Ca,Ga-modified catalysts **(b)**. 3-MP cracking conditions: $T = 400$ °C, $m_{cat} = 20$ mg (150–212 µm), 1 bar, carrier gas N_2 = 50 mL/min, 3-MP partial pressure = 3.8 kPa in the presence of 2,4-dimethylquinoline (<0.1 kPa) to prevent the cracking over BAS at the external catalyst surface[2], $P_{H2O} = 0.6$ kPa. The conversion and product selectivity are averaged values within TOS = 0.1–0.4 h.

S2. Supplementary Computational Results

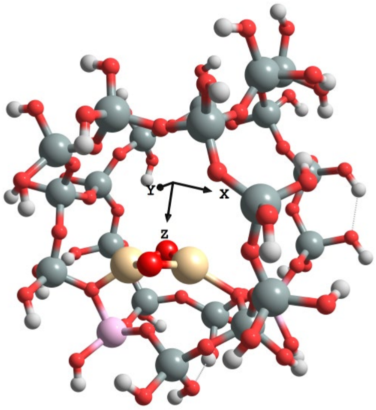

a. Structure $Ga_2O_2^{2+}$ confined in alpha site

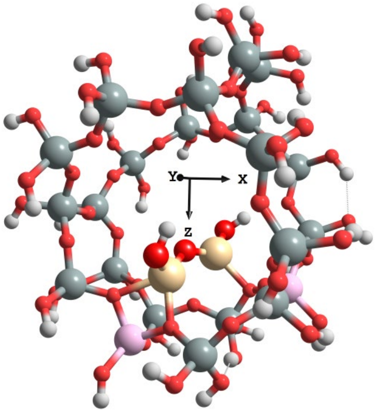

b. Structure $Ga_2O_2(H_2O)^{2+}$ confined in alpha site

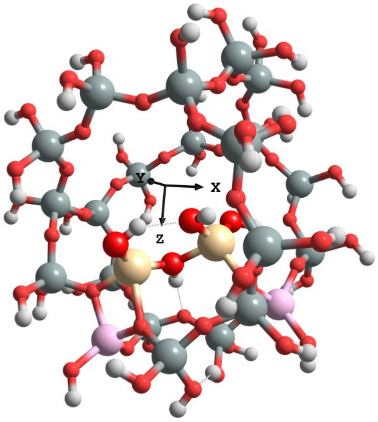

c. Structure $Ga_2O_2(H_2O)_2^{2+}$ confined in alpha site

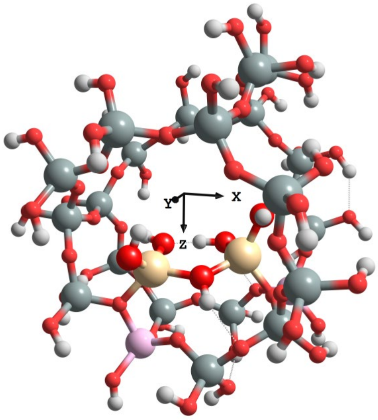

d. Structure $Ga_2O_2(H_2O)_3^{2+}$ confined in alpha site

Figure S6. Global minima of the low hydrated $Ga_2O_2(H_2O)_x^{2+}$ confined in the alpha site of the ZSM-5 and optimized at PBE-D3(BJ) level of theory, where x = 0(a), 1(b), 2(c), 3(d). Aluminum is pink, oxygen is red, gallium is beige, silicon is grey, hydrogen is white.

***Alpha* site in ZSM-5**	***Beta* site in ZSM-5**	***Gamma* site in ZSM-5**

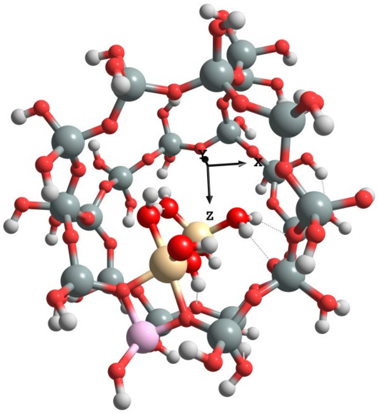

a. Structure $Ga_2O_2(H_2O)_4^{2+}$confined in alpha site

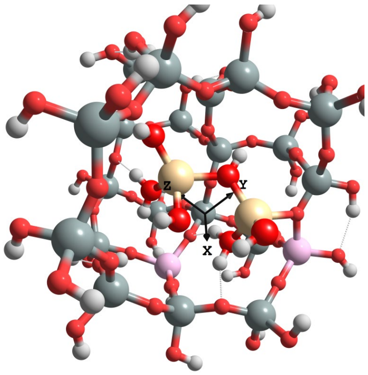

b. Structure $Ga_2O_2(H_2O)_4^{2+}$ confined in beta site

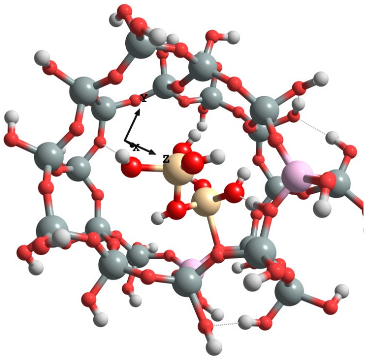

c. Structure $Ga_2O_2(H_2O)_4^{2+}$ confined in gamma site

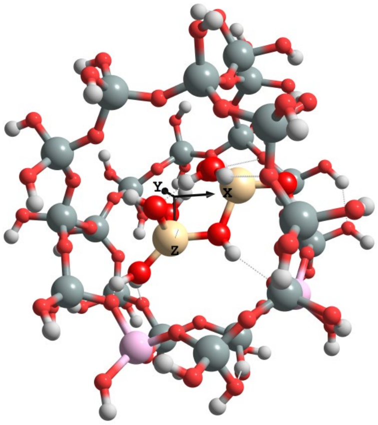

d. Structure $Ga_2O_2(H_2O)_5^{2+}$confined in alpha site

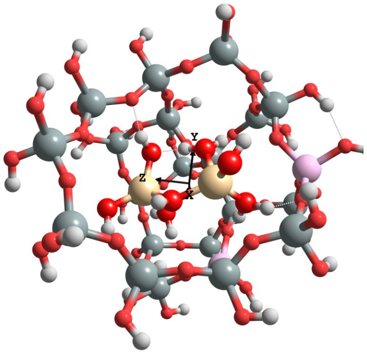

e. Structure $Ga_2O_2(H_2O)_5^{2+}$ confined in beta site

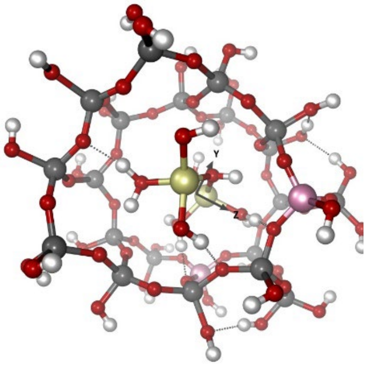

f. Structure $Ga_2O_2(H_2O)_5^{2+}$ confined in gamma site

Figure S7. Global minima of $Ga_2O_2(H_2O)_x^{2+}$ confined in the alpha (column 1: a, d), beta (column 2: b, e), gamma (column 3: c, f) sites of the ZSM-5 and optimized at PBE-D3(BJ) level of theory, where x = 4(a, b, c), 5(d, e, f). Aluminium is pink, oxygen is red, galium is beige, silicon is grey, hydrogen is white.

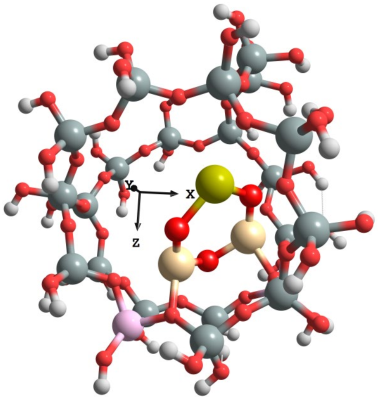

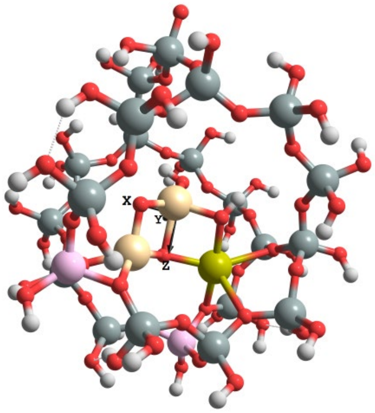

a. Structure $CaGa_2O_3^{2+}$ confined in alpha site

b. Structure $CaGa_2O_3(H_2O)^{2+}$ confined in alpha site

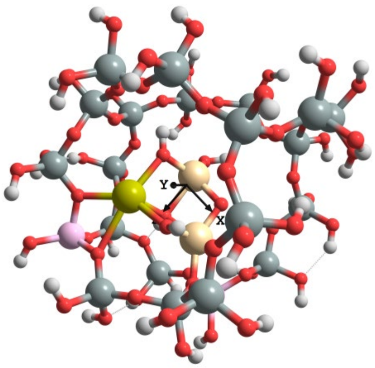

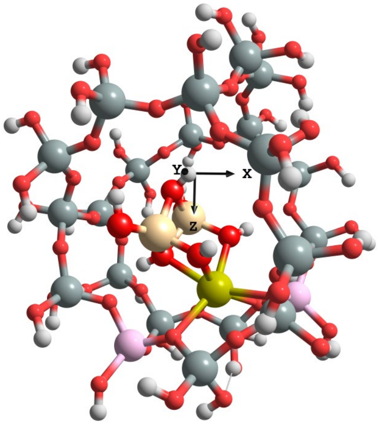

c. Structure $CaGa_2O_3(H_2O)_2^{2+}$ confined in alpha site

d. Structure $CaGa_2O_3(H_2O)_3^{2+}$ confined in alpha site

Figure S8. Global minima of the low hydrated $CaGa_2O_3(H_2O)_x^{2+}$ confined in the alpha site of the ZSM-5 and optimized at PBE-D3(BJ) level of theory, where x = 0(a), 1(b), 2(c), 3(d). Aluminum is pink, oxygen is red, gallium is beige, silicon is grey, hydrogen is white, calcium is yellow.

Alpha **site in ZSM-5** | ***Beta*** **site in ZSM-5** | ***Gamma*** **site in ZSM-5**

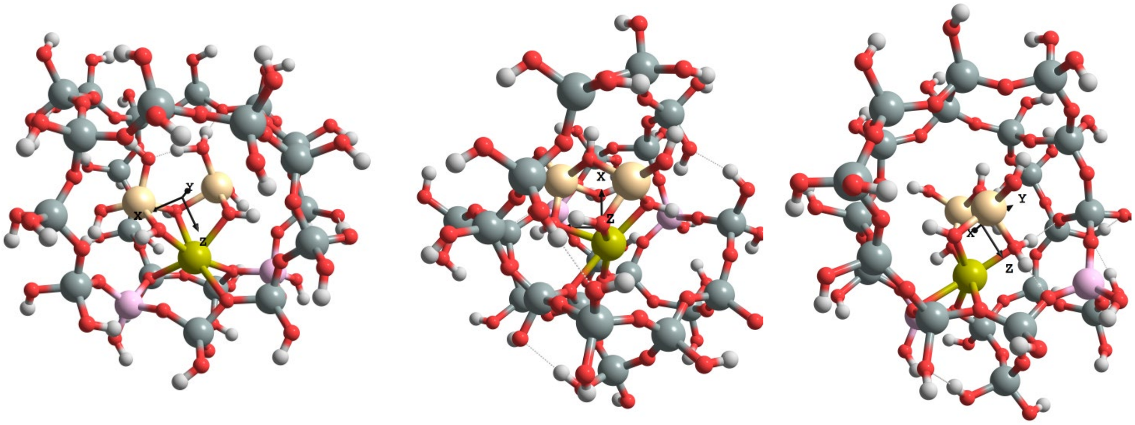

a. Structure $CaGa_2O_3(H_2O)_4^{2+}$confined in alpha site

b. Structure $CaGa_2O_3(H_2O)_4^{2+}$ confined in beta site

c. Structure $CaGa_2O_3(H_2O)_4^{2+}$ confined in gamma site

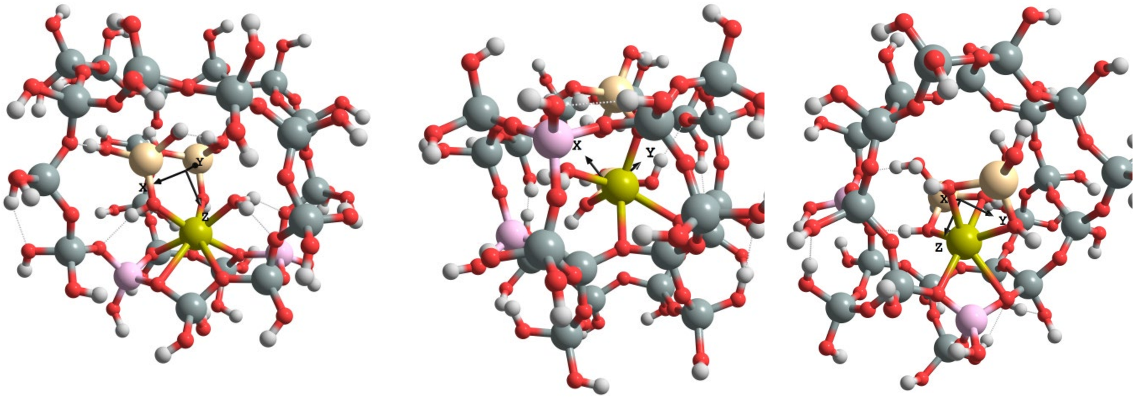

d. Structure $CaGa_2O_3(H_2O)_5^{2+}$confined in alpha site

e. Structure $CaGa_2O_3(H_2O)_5^{2+}$ confined in beta site

f. Structure $CaGa_2O_3(H_2O)_5^{2+}$ confined in gamma site

Figure S9. Global minima of $CaGa_2O_3(H_2O)_x^{2+}$ confined in the alpha (column 1: a, d), beta (column 2: b, e), gamma (column 3: c, f) sites of the ZSM-5 and optimized at PBE-D3(BJ) level of theory, where x = 4(a, b, c), 5(d, e, f). Aluminium is pink, oxygen is red, gallium is beige, calcium is yellow, silicon is grey, hydrogen is white.

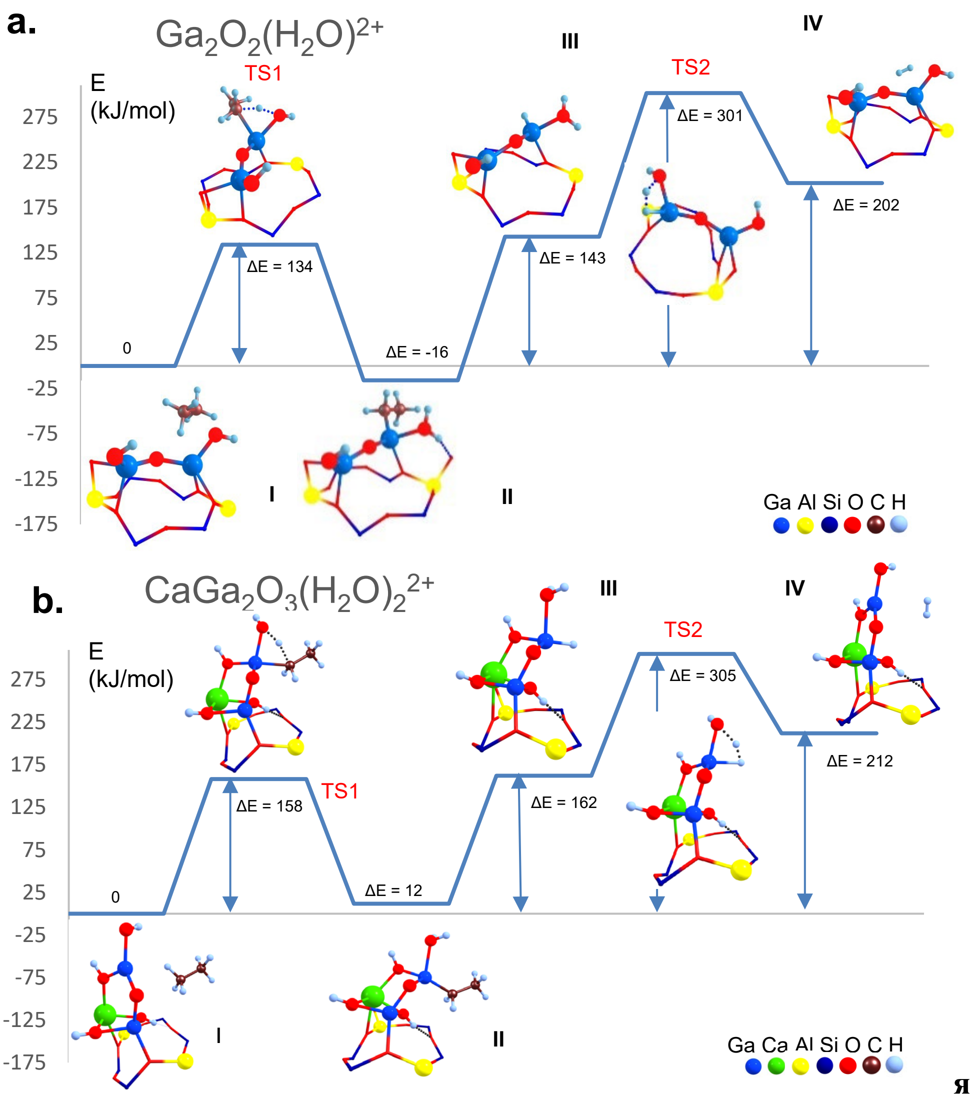

Figure S10. DFT-computed reaction energy diagrams and local optimized structures of the key intermediates and transition states for ethane dehydrogenation over (a) $Ga_2O_2(H_2O)^{2+}$ (b) and $CaGa_2O_3(H_2O)_2^{2+}$ clusters in ZSM-5 zeolite thermodynamically favoured at low H_2O partial pressures (-2.0 eV < $\Delta\mu_{H2O}$ < -1.2 eV).

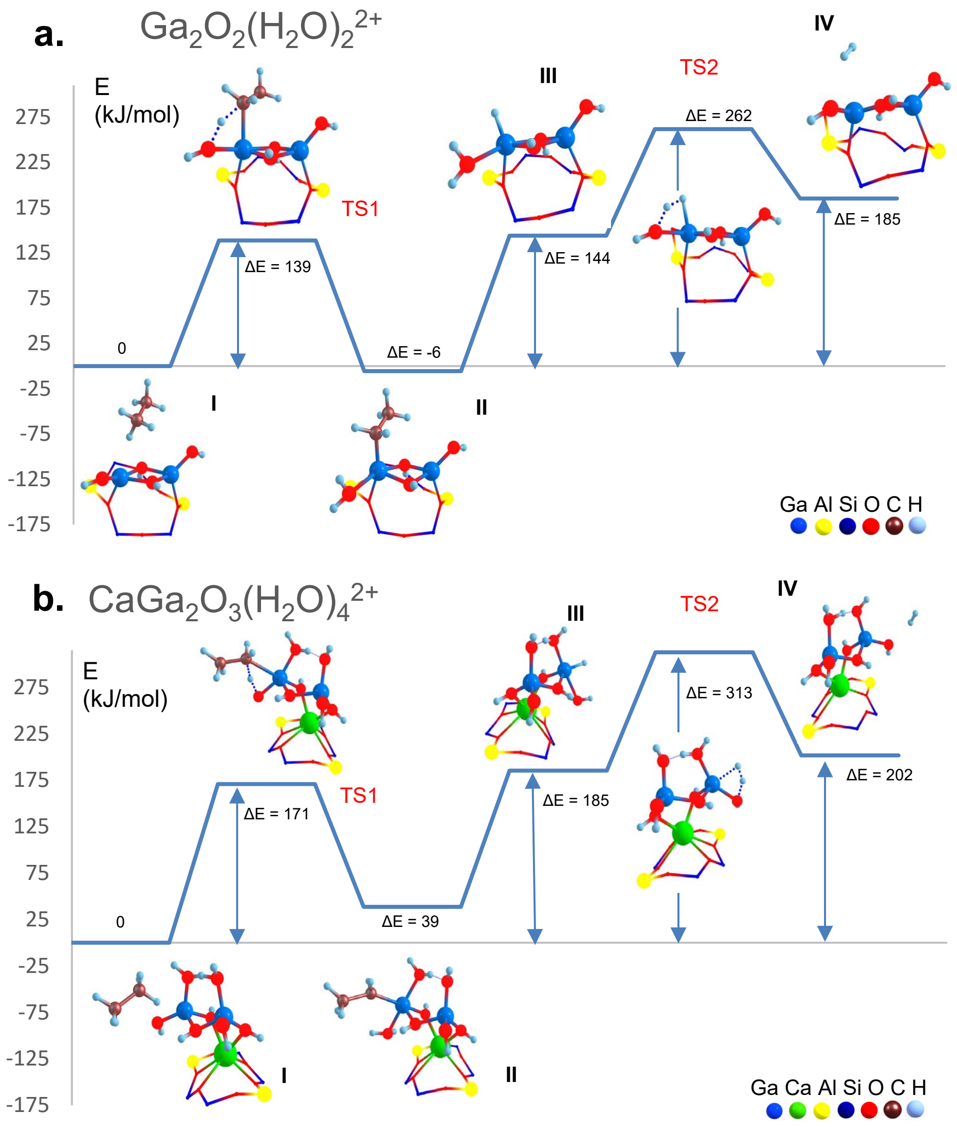

Figure S11. DFT-computed reaction energy diagrams and local optimized structures of the key intermediates and transition states for ethane dehydrogenation over (a) $Ga_2O_2(H_2O)_2^{2+}$ and (b) $CaGa_2O_3(H_2O)_4^{2+}$ clusters in ZSM-5 zeolite thermodynamically favoured at H_2O partial pressures relevant to the MTA process ($\Delta\mu_{H2O} > -1.2$ eV).

Table S3. The reaction energies (ΔE) and the activation barriers ($E_{act}^{‡}$) of the C-H-bond activation, β-elimination and H_2 recombination steps of ethane dehydrogenation.

**Reaction step**	**C-H-bond activation**		**β-elimination**		**H_2 recombination**	
**Structures**	ΔE	$E_{act}^{‡}$	ΔE	$E_{act}^{‡}$	ΔE	$E_{act}^{‡}$
**$Ga_2O_2(H_2O)^{2+}$**	-16	134	159	/	59	158
**$Ga_2O_2(H_2O)_2^{2+}$**	-6	139	150	/	41	118
**$CaGa_2O_3(H_2O)_2^{2+}$**	12	158	150	/	50	143
**$CaGa_2O_3(H_2O)_4^{2+}$**	39	171	147	/	16	128

Supplementary References

1. Gabrienko, A. A.; Danilova, I. G.; Arzumanov, S. S.; Pirutko, L. V.; Freude, D.; Stepanov, A. G., *The Journal of Physical Chemistry C* **2018,** 122 (44), 25386-25395.
2. Yokoi, T.; Mochizuki, H.; Namba, S.; Kondo, J. N.; Tatsumi, T., *The Journal of Physical Chemistry C* **2015,** 119 (27), 15303-15315.
3. Benco, L.; Bucko, T.; Hafner, J.; Toulhoat, H., *The Journal of Physical Chemistry B* **2004,** 108 (36), 13656-13666.
4. Pidko, E. A.; Hensen, E. J. M.; van Santen, R. A., *The Journal of Physical Chemistry C* **2007,** 111 (35), 13068-13075.
5. Pidko, E. A.; Hensen, E. J. M.; Zhidomirov, G. M.; van Santen, R. A., *Journal of Catalysis* **2008,** 255 (2), 139-143.
6. Katz, A. K.; Glusker, J. P.; Beebe, S. A.; Bock, C. W., *Journal of the American Chemical Society* **1996,** 118 (24), 5752-5763.
